# Erythrocyte’s aging in microgravity highlights how environmental stimuli shape metabolism and morphology

**DOI:** 10.1038/s41598-018-22870-0

**Published:** 2018-03-27

**Authors:** S. Dinarelli, G. Longo, G. Dietler, A. Francioso, L. Mosca, G. Pannitteri, G. Boumis, A. Bellelli, M. Girasole

**Affiliations:** 1 Istituto di Struttura della Materia – CNR, Via fosso del cavaliere 100, 00133 Roma, Italy; 2 LPMV–IPhys-EPFL, Route de la Sorge, Lausanne, Switzerland; 3 Dipartimento di Scienze Biochimiche “A. Rossi-Fanelli” Universita “Sapienza”, Piazzale A. Moro 5, 00185 Roma, Italy; 4 Dipartimento di Scienze cardiovascolari, respiratorie, nefrologiche, anestesiologiche e geriatriche Università “Sapienza”, Piazzale A. Moro 5, 00185 Roma, Italy

## Abstract

The determination of the function of cells in zero-gravity conditions is a subject of interest in many different research fields. Due to their metabolic unicity, the characterization of the behaviour of erythrocytes maintained in prolonged microgravity conditions is of particular importance. Here, we used a 3D-clinostat to assess the microgravity-induced modifications of the structure and function of these cells, by investigating how they translate these peculiar mechanical stimuli into modifications, with potential clinical interest, of the biochemical pathways and the aging processes. We compared the erythrocyte’s structural parameters and selected metabolic indicators that are characteristic of the aging in microgravity and standard static incubation conditions. The results suggest that, at first, human erythrocytes react to external stimuli by adapting their metabolic patterns and the rate of consumption of the cell resources. On longer timeframes, the cells translate even small differences in the environment mechanical solicitations into structural and morphologic features, leading to distinctive morphological patterns of aging.

## Introduction

Erythrocytes, or Red Blood Cells, (RBCs) are interesting biosystems for a variety of reasons, the most remarkable being their physiological role as exclusive O_2_ and CO_2_ transporters in the body. They have peculiar characteristics, such as absence of DNA and simplified structure and metabolism that suggests that these cells have a special relationship with the environment. Overall, external stimuli effectively act as a modulators of RBCs shape and function^1^. In particular, environmental mechanical solicitations can regulate behaviour and structural properties of the cell through various mechanisms including the mechanotransduction-mediated expulsion of ATP^2,3^.

In the human body, the maintenance of a constant number of RBCs in the blood is the result of a dynamic equilibrium between the production of precursors in the bone marrow and the removal of senescent cell, which makes the aging a fundamental regulative phenomenon. Indeed, aging degrades the characteristics of the circulating RBCs in a donor-dependent fashion^4^ but also critically influences the blood homeostasis. Since the *in vivo* removal of senescent or pathological cells is triggered by the occurrence of morphological anomalies on the cell surface (most of which are mediated by cytoskeleton disorders) quantitative microscopy techniques such as Atomic Force Microscopy, are ideal to characterize the aging pathways or the role of blood pathologies^5,6^.

In addition to the study of aging, there has been an increasing interest towards the understanding of the biochemical and structural alterations that occur when biosystems, and in particular cells, are maintained in zero-gravity or microgravity conditions. This interest is certainly stimulated by the increasing human activities on the international space station (ISS) and in foreseeable long-term space missions. Indeed, there have been many scientific evidences of alterations occurring in cellular systems (e.g. osteoblasts, cells of the immune system, chondrocytes, cells of the muscles, stem or cancer cells)^7–10^ exposed to extra-terrestrial environment. Furthermore, it has been shown that microgravity affects the genetic expression, differentiation and the organization of cytoskeletal actin, thus producing transient or permanent effects on the cell morphology (rounding)^11,12^. Some of the complications experienced during the permanence in space can have a clinical interest. Microgravity causes atrophy of the muscular system, dysfunction of the immune system, electrolyte imbalance and cardiovascular anomalies and reduction in bone mass and osteopenia^13–15^.

Concerning RBCs, there are reports of space-flight-induced oxidative stress^16^, in which the direct role of microgravity is still in discussion, and there are several evidences of blood homeostasis alterations such as the so called space anaemia^17^, which leads to a transient increase of haematocrit and Hb concentration, counteracted by destruction of immature RBCs in the bone marrow^18–20^. The space-anaemia, and the relative neocytolysis, can be a suitable model for pathological situations involving renal failure and decrease of circulating erythropoietin, but also for para-physiological conditions in which altitude-acclimated subjects are rapidly moved to sea level^21,22^.

For sure, gravitational biology is strongly related to the mechanobiology^23^ and, in this sense, studies involving RBCs are particularly intriguing, because of their efficient biomolecular machinery dedicated to the sensing of external forces and to their translation into biological activity. Unfortunately, up to now little is known about the relationship between metabolic adaptations induced by microgravity and their structural or morphological characteristics, nor has the fundamental pattern of aging been investigated^24^.

The increasing demand for experiments in space has stimulated the development of technologies to deliver nearly zero-gravity environmental conditions on Earth^25^. Simulated microgravity requires a functional near-weightlessness and, quantitatively, any system capable to deliver gravity between 10^−3^ and 10^−6^ g can be employed to simulate ISS-like space conditions^26,27^. The most employed methods to deliver microgravity are random positioning machines (RPM) and 3D-clinostats. In these instruments, the samples undergo fast rotations over the three axes, resulting in an average null gravity vector. The difference between these two systems consists in the rotation speed, which is constant in the 3D-clinostat and involves randomly oriented accelerations in the RPM. Both tools have been validated by international space agencies (including ESA) and the choice of the best methodology depends on the cellular characteristics and, for cells in suspension such as RBCs, the use of the 3D-clinostat results preferable^12,28^.

Here we present an approach integrating different biochemical and biophysical techniques to thoroughly evaluate the structure and function of RBCs comparing the aging patterns in normal- and in 3D-clinostat-delivered microgravity conditions.

Overall, this work provides the basis to understand how RBCs sense microgravity and transduce this stimulus into metabolic, structural and morphologic patterns.

## Results

### Monitoring the stress markers along the RCSs’ aging

We followed the stresses accumulated into the RBCs throughout the aging pathway. Specifically, we considered the time evolution of the rate of cells lysis and the oxidation state of the haemoglobin (Hb). This is of utmost importance since Hb denaturation plays a key role in correlating the functional to the structural alterations of the cells along the ageing^29,30^.

**Figure 1:**
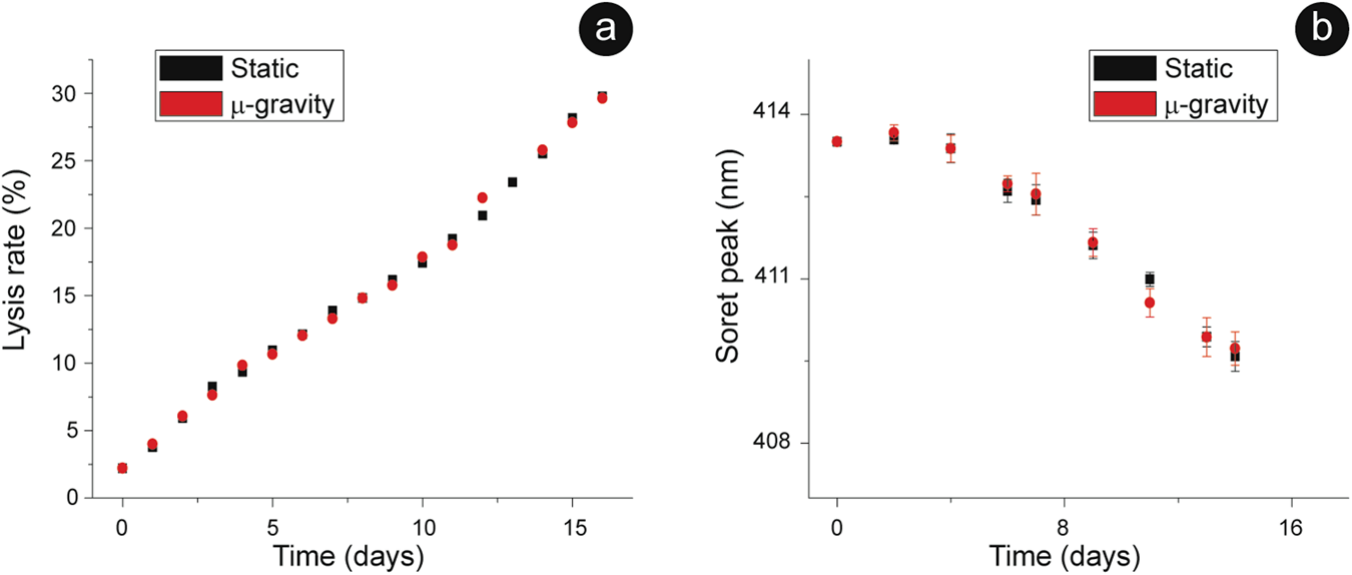
Biochemical assays over the aging pathway. Biochemical stresses experienced by the cells along the aging monitored through the overall rate of cell lysis (**a**) and the intracellular Hb oxidation (**b**). Both stress indicators follow an equivalent time evolution in static and microgravity aged RBCs. The error bars (SD) are reported in both graphs.

We quantified the cell lysis and the oxidation state of the intracellular Hb by monitoring the Soret peak (414 nm in the oxy-Hb while shifted towards a lower wavelength in the met-Hb), at different aging times for RBCs in static and in microgravity conditions (Fig. 1). The results evidence that, as time passes, the environmental stimuli become progressively heavier and, consequently, there was an increase of both the total lysis and the ferric character of Fe-Hb. Remarkably, the trends for the static and microgravity-exposed samples are practically identical. The nearly linear time course of the lysis (Fig. 1a) is consistent with the hypothesis of an accelerating process of biochemical deterioration.

### Effect of rejuvenation treatments

To gain a better insight on the real similarities between these samples and to understand some metabolic strategies in stress conditions, we incubated the RBCs, at given time-points throughout the ageing, in the presence of precursors that allow to rebuild the cellular resources. In this rejuvenation process, only the metabolically active cells can recover their resources. Thus, the process somehow re-defines the T0 and intrinsically selects the residual fraction of active cells.

**Figure 2:**
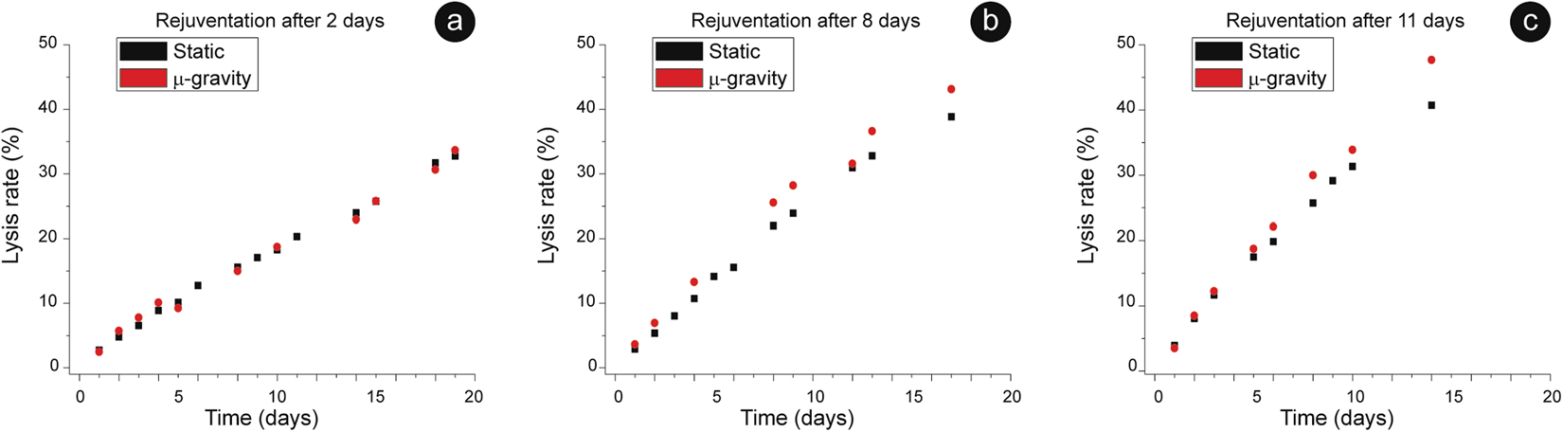
Cell lysis upon rejuvenation. Trends of RBCs lysis observed after revitalization performed, respectively, at day 2 (panel a); day 8 (**b**) and day 11 (**c**). In the three panels, the 0 was set at the time of rejuvenation and the plots report the lysis observed in the following days. In RBCs rejuvenated after 8 (**b)** and 11 (**c**) days of aging, some differences emerge: the microgravity-exposed cells always behave as the more stressed samples i.e. they show higher lysis rate than in the static.

The lysis patterns in the days that follow each revitalization are shown in Fig. 2. Clearly, the, cells revitalized after 2 days showed a lysis pattern identical to the un-revitalised samples, and this was independent on the static or microgravity environment (Fig. 2a). The same result was observed after revitalization at day 4 (data not shown). On the contrary, samples rejuvenated after 8 days or more, had lysis rates after rejuvenation higher than un-revitalized samples. Thus, in both environments, between the 4th and 8th day of aging the RBCs appeared to develop peculiar alterations that made the cells weaker and more fragile.

More interestingly, since after day 8 the revitalized microgravity cells had a lysis rate higher than the corresponding static erythrocytes (see Fig. 2b or c), the aging in microgravity delivered larger stress to the cells compared to the static incubation.

**Figure 3:**
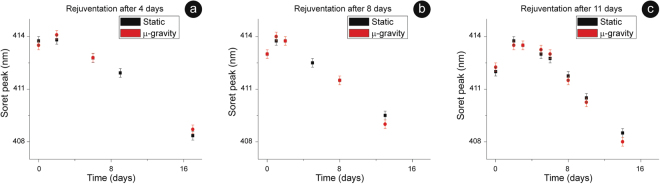
Hb oxidation upon rejuvenation. Kinetic of Hb’s re-oxidation after a revitalization performed at day 4 (panel a), at day 8 (**b**) or at day 11 (**c**) of aging. In the panels, the 0 was set at the time of rejuvenation and the plots report the behaviour observed in the following days. Revitalization provides a source of freshly synthetized redox power that is spent, in the first days after rejuvenation, to ensure an extra-reduction of the Hb. The magnitude of this reduction increases with the aging: for instance, in microgravity, goes from 0,5 nm in panel (**a**) to 0,75 nm in (**b**) and to 1,75 nm in (**c**), indicating that an increased amount of reducing power is spent to this aim. The data point for the two samples are, sometimes, identical while the reported error bar is 0,25 nm.

Concerning the intracellular Hb, whose data are summarized in Fig. 3, we observed little or no differences in the aging patterns between static and microgravity oxidation, at least up to the rejuvenation performed after 11 days. Interestingly, in both environments, the 24/48 hours that followed a revitalization were characterized by an extensive reduction of the Hb. The efficiency of this phenomenon increased with the revitalization time: it was smaller for cells revitalized after 2 or 4 days and it became progressively higher for RBCs rejuvenated at the 8th or at the 11th day. After the complete reduction of the Hb (that always led back to, approximately, 414 nm), the oxidation pattern restarted.

This behaviour suggests that the priority in the use of the redox power is the reduction of the Hb.

### Monitoring the cell resources along the aging

**Figure 4:**
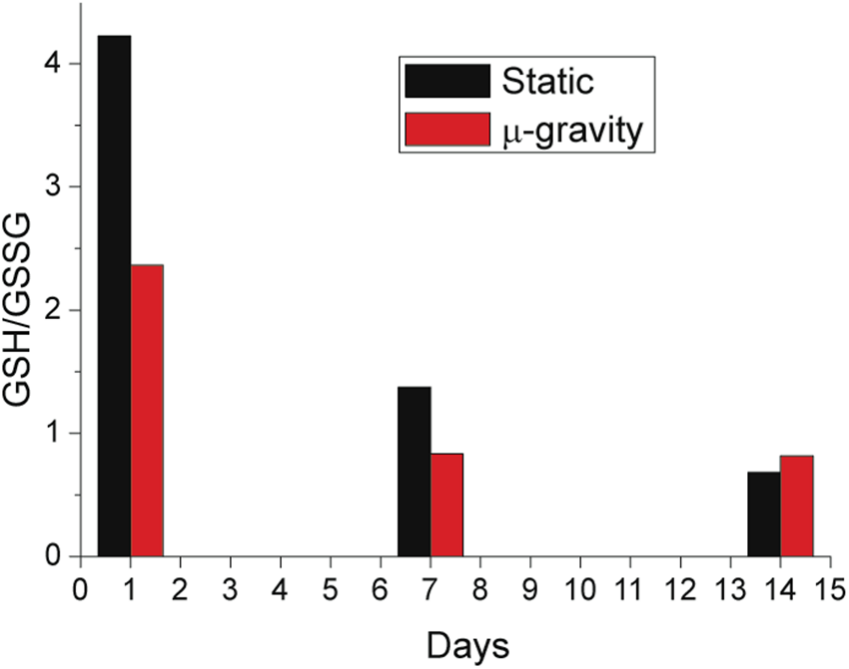
Cell redox power. The GSH/GSSG ratio measured for the microgravity (red) and static sample (black). In microgravity, the consumption of GSH is much faster than in static RBCs. Furthermore, as a GSH/GSSG ratio below 1 indicates that the reducing power is at the basal level, the protective effect of the reducing power lasts longer in static conditions than in microgravity (see the data after 7 days).

We report, in Fig. 4, the measurement of the cells’ redox state at different aging times for the samples maintained in static and microgravity conditions. The measurements were performed by quantifying the ratio between the reduced and oxidized forms of glutathione (GSH/GSSG), which must remain above 1 to ensure a proper reducing environment.

The kinetics of the redox states observed in static and microgravity samples are clearly different.

In particular, in microgravity, the consumption of redox power was much faster, especially in the first day. Furthermore, the protecting role of glutathione in microgravity lasted for a shorter time: after 7 days, the reducing power in static cells was weak but still present, while in microgravity the GSH/GSSG ratio was already below 1. These results suggest that, compared to static conditions, the RBCs in microgravity underwent environmental effects that required a higher consumption of GSH to maintain a proper cell function.

As the synthesis of reducing power in RBCs is linked to the ATP metabolism, we monitored also this energy resource along the aging pathway.

**Figure 5:**
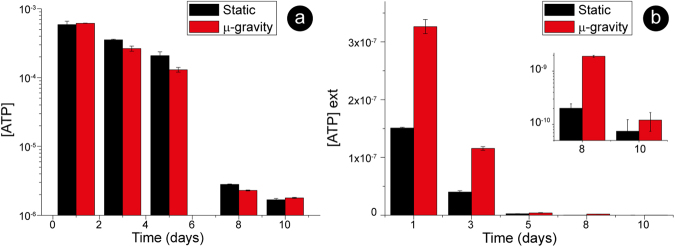
ATP throughout the aging pathway. Intracellular (panel a, log scale) and extracellular ATP (panel b, linear scale) measured after 1, 3, 5, 8 and 10 days. The intracellular ATP in static and microgravity conditions follows a similar trend, although there is a faster consumption in microgravity (note the logarithmic scale). The pattern of ATP expulsion (panel b) indicates, at least for the first 8 days, very different regulations for this phenomenon in the two samples.

The data of intracellular ATP reported in Fig. 5a evidences that the ATP consumption in microgravity was faster than in the static samples, especially during the first 5 days. Furthermore, the intracellular ATP concentration exhibited a sharp, threshold-like, change during storage that took place between 5 and 8 days of aging. This had already been observed^31^ and is most likely due to a strong acceleration in the ATP consumption.

The expulsion of ATP is part of an important physiological response to mechanical stimulation^3,32–34^. After the expulsion, the extracellular ATP is subject to a dynamic balance between the expelled and the consumed molecule. Indeed, the ATP hydrolysing enzymes (ecto-nucleotidases) have a constant, and relatively slow, kinetics of hydrolysis that allows measuring the ATP expelled by the cells, essentially, in the last 20–30 minutes^35^. The measurement of the ATP in the extracellular medium (Fig. 5b) evidenced that the release pattern was markedly different: RBCs aged in microgravity expelled much more ATP compared to static cells. Therefore, the best interpretation of our data is that, during the aging, the erythrocytes feel different mechanical stimulations in microgravity or static condition.

### Morphological and morphometric patterns

**Figure 6:**
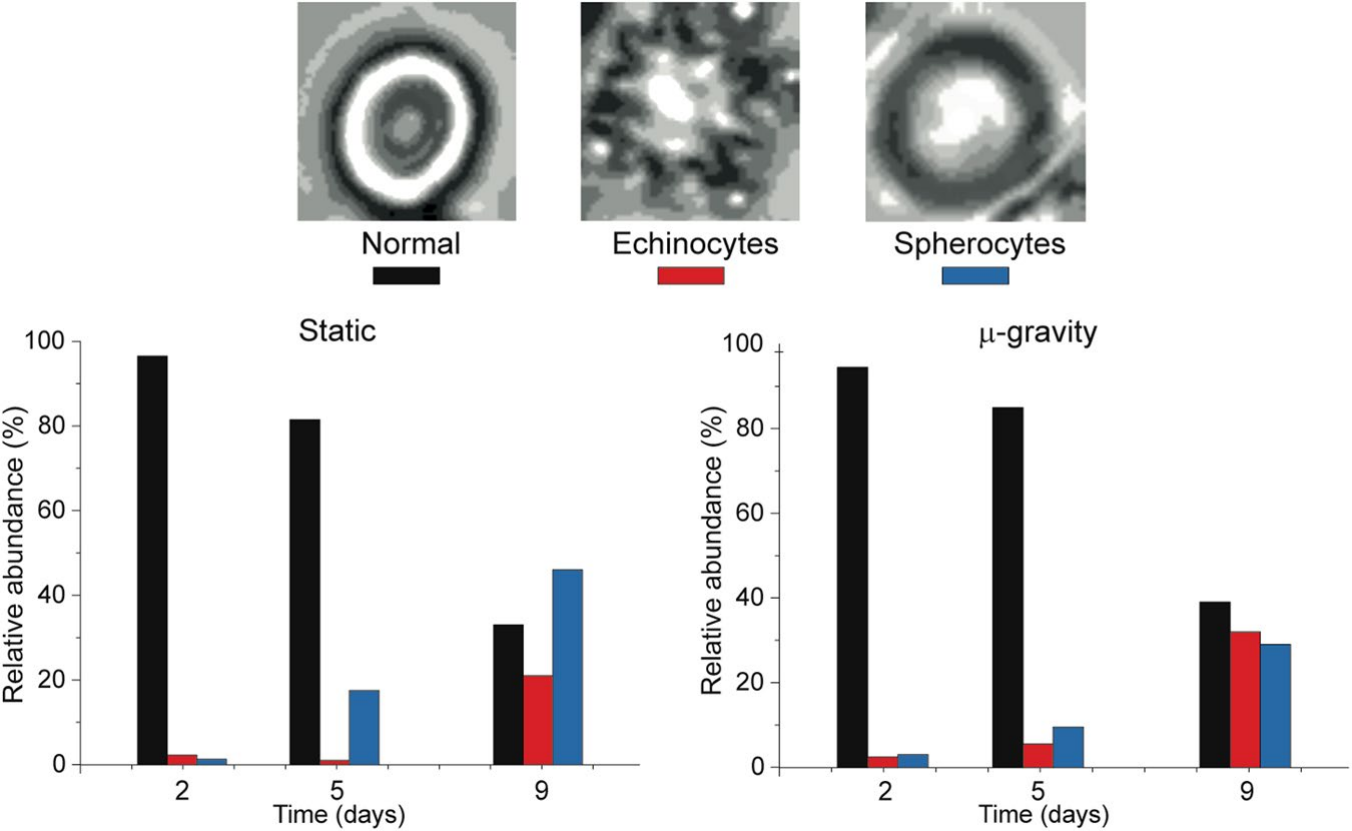
Morphological evolution. Morphological patterns observed along the aging in static and microgravity conditions. The two samples are equivalent after short incubations (i.e. 2 days) but, depending on the static or microgravity environment, the pattern of aging differs: in microgravity there is no preference between spherocytes and echinocytes while in static condition the morphological evolution clearly privileges the occurrence of spherocytic RBCs. The histograms were built by analysing at least 1000 cells per sample.

We evaluated, through conventional and high-resolution microscopy, the structural and morphological consequences of the environmental stimulations. We used optical microscopy to determine the overall RBCs’ shape after 2, 5 and 9 days in static or microgravity conditions, while at the same time monitoring the membrane roughness by AFM (Fig. 6).

For each analysed sample, we divided the cells into three basic categories that represent the most important morphological “phenotypes” that can be encountered along the aging path: (1) no significant alterations (biconcave or slightly crenated); (2) spiculed cells (echinocytes) and (3) spherocytes. We limited our study to these three categories due to the intrinsic limitations of conventional microscopy, yet the observed aging pattern was still quite meaningful. Indeed, the samples incubated in the two conditions were identical at t = 0 and were still very similar after two days. At higher aging times, distinct morphological evolutions appeared: in the static samples, the dominant anomalies after 5 and 9 days were the spherocytic cells, while, in microgravity, echinocytes and spherocytes were present in equivalent amounts (i.e. with a sharp increase of the spiculed morphology that is usually considered a more severe marker of aging).

**Figure 7:**
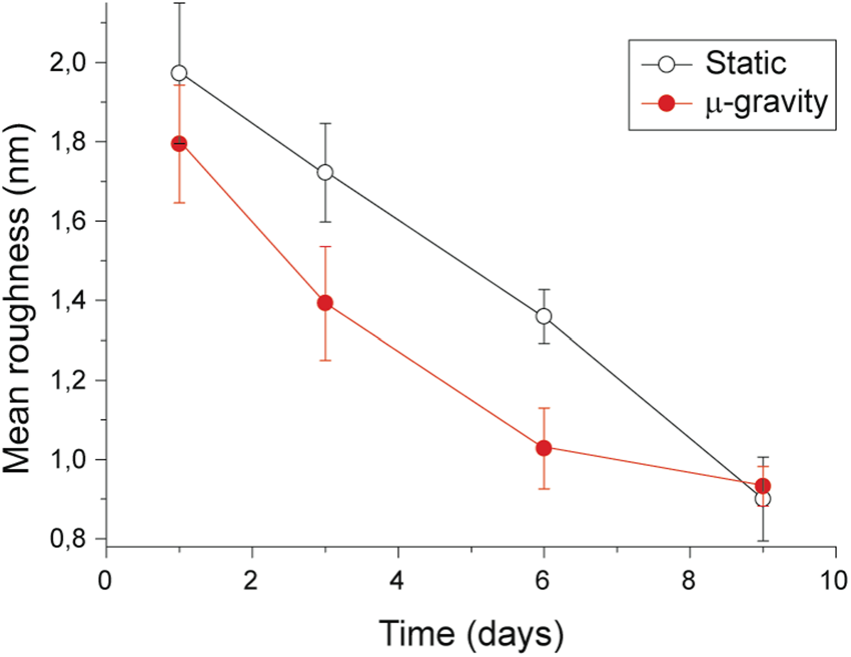
Quantitative morphological data. Mean membrane roughness measured along the aging for static (hollow symbols) and microgravity (solid symbols) incubated cells, with these latter showing a faster decrease in the roughness.

The membrane roughness data (Fig. 7) presented a continuous decrease at increasing aging times that can be interpreted on the basis of the progressive reduction of the mechanical support exerted by the cellular skeleton. This decreasing trend was considerably faster in microgravity, compared to the static samples, indicating that in microgravity the RBCs translate faster the environmental stimuli into a structural weakening. Despite that, the final state observed, after 9 days of aging, is the same in both experimental conditions.

As a whole, the morphological data (Figs 6 and 7) revealed different landscapes that depend on the specific environmental conditions. In detail, the microgravity-exposed cells showed, at increasing aging times, more severe morphological defects and a faster occurrence of structural weakening and alterations.

## Discussion

In this study, we focused on the strategies employed by RBCs to sense and adapt to an environmental stimulation and on the mechanisms these cells use to translate metabolic regulation into specific morphological patterns.

We monitored the aging path of the RBCs combining the time-related variations of several parameters:

- rate of cell lysis and Hb oxidation state to evaluate the biological stress;
- redox power (e.g. the GSH/GSSH ratio) and the intracellular and extracellular ATP, to determine the cell’s resources and the metabolic status;
- cell shape and membrane roughness, as morphological and structural indicators.

These parameters are, in different ways, correlated. For example, to maintain the reduced state of the Hb’s iron, which is fundamental to ensure the reversible binding of oxygen, RBCs must consume high quantities of reducing power, thus influencing the cell’s redox state. The synthesis of ATP and reducing power (NADPH) can be shunted through the pentose phosphate pathway, in order to produce the first or the second according to the cell needs. Also, the morphological parameters are related to the biochemical and metabolic data through the integrity and arrangement of the membrane-skeleton, resulting in variations of the membrane roughness. Aging leads to quantitatively relevant reduction of the roughness^36^ which has been associated to shortage of ATP^37–39^.

With these premises, we performed several analyses to understand some of the RBCs’ metabolic strategies exerted under stress and comparing their time-dependent evolution in static or microgravity conditions. In both environments, as the aging increases, the observed landscape was indicative of a progressive loss of the cellular function, coupled with a degradation of RBCs’ structures.

Comparing the aging pathways in static and microgravity conditions revealed close analogies in the lysis rate and Hb oxidation. On the other hand, it evidenced a different management of the cell resources: in microgravity, we measured a faster consumption of reducing power and of intracellular ATP, coupled to an acceleration of the pathways leading to ATP expulsion compared to the static counterparts (Figs 4 and 5). Overall, we can propose that, given the relationship between the parameters in the presence of the different environmental stimulations, the RBCs modulate their metabolic pathways in order to maintain a standard rate of cell lysis and Hb oxidation.

Remarkably, we also highlighted that the biochemical and morphological responses to the different environmental conditions take place in distinct time scales: a faster biochemical adaptation followed by slower morphological alterations.

Indeed, most of the differences in the redox state and extracellular ATP between the cells kept in static and microgravity conditions (Figs 4 vs 5b) occurred in the first days of aging, when the metabolic adaptive mechanisms were clearly different. In the first two-three days, the Hb oxidation did not occur (Fig. 1), suggesting that the consumption of GSH in the two environments should be equivalent. Yet, there was a sharp difference in the measured reducing powers (Fig. 4) that indicated the existence of an additional environmental stimulation in microgravity

Another very evident effect of microgravity on the cells was a much larger expulsion of ATP compared to static cells. This is of the utmost importance since the most physiologically relevant causes for ATP expulsion in RBCs *in vivo*^32^ are the mechanical stimulations produced by the narrow capillaries that permit the normal flow of RBCs^2,3,34,40^. Thus, we can conclude that the RBCs in microgravity are exposed to a higher mechanical stimulation compared to the static counterpart. Thus, different environmental mechanical stimulations can be assumed as the driving forces that, at least partially, determine a different consume of the cell resources.

The different metabolic control in static and microgravity conditions was also reflected in the morphology and morphometric patterns, but on a longer time scale. For instance, the trend of the membrane roughness in microgravity (Fig. 7) showed, in the medium time scale (roughly, 3–9 days), a faster decrease of the skeletal structural support (i.e. a faster aging) compared to the static incubation^36^. This faster cell aging was also confirmed by the analysis of the overall RBCs’ shape patterns (Fig. 6). Indeed, the microgravity sample had larger abundance of cells with morphologies correlated to more advanced aging (i.e. echinocytes)^41^ compared to the static incubation (dominated by spherocytes).

A central role in the development of such morphological defects is certainly played by the membrane-skeleton properties. In addition to the roughness decrease associated to a shortage of ATP, we observed that the abnormal shapes (e.g. echinocytes) resulting from the loss of skeletal control, grew strongly in the very same time-frame in which the consumption of ATP sharply increased (5–9 days), leading to its substantial exhaustion. This suggests that ATP could be the most effective biochemical factor governing the RBCs morphology and its deficiency could pave the way, through the involvement of the cytoskeleton, to the onset of morphological anomalies^38,42–45^. On the other hand, our data show that the redox power has the priority to maintain the Hb in the reduced state. For instance, after rejuvenation performed at long aging times (8 or 11 days), a full recovery of the ATP and GSH preludes to a significant effort in the Hb reduction (Fig. 3). On the contrary, previous experiments in static conditions, demonstrated that this doesn’t occur for the ATP-dependent morphometric parameters (e.g. the roughness)^31^.

This behaviour suggests what the cell priorities in shortage of resources are: when recovering the full metabolic potential, the cells privilege the exploitation of the redox power to maintain the Hb in a reduced state, rather than a full recovery of the membrane-skeleton structure.

Focusing on the cell resources, the rates of GSH and ATP production are correlated^46^, yet their consumption appears certainly independent. In particular, they respond differently to the stress induced by microgravity compared to the static incubation. Specifically, the higher mechanical stress delivered in microgravity, results in an increased consumption, on different time frames (Figs 4 vs 5), of both ATP and GSH. While the molecular mechanisms that translate the microgravity solicitation into a decrease of ATP and GSH have not yet been clarified, it is the metabolic interplay between these two effectors that determines long-term structural effects on the cell.

In this framework, a few considerations on the nature of the higher stimulation observed in microgravity are in order.

The cells aged in static conditions experience a modest and steady pressure on their membrane, exerted by the surrounding cells, while those aged in 3D-clinostat delivered microgravity feel a gravity vector constant in modulus but with a direction continuously variable over time, in such a way that its average is zero over a typical time-frame. Besides this modulated stimulation acting point by point on the entire membrane, in simulated microgravity the RBCs are dragged into a pseudo-spherical motion with scale size comparable to the cell dimensions, and practically determined by the physico-chemical characteristics of samples and buffer.

While the aim of the present study was to provide a descriptive overview of complex phenomena and of their major correlations, further studies are currently in progress to better understand some causal relationships only marginally discussed here. These include the effect of microgravity on the peroxidation of membrane lipids and the identification of the molecular targets for the mechanical/environmental stimulation that trigger the cell metabolism. Also the nature of the biochemical effectors that translate the metabolic regulation into morphometric pattern need to be better defined in future studies.

In conclusion, as a result of a study performed with an unprecedented variety of techniques and methodologies, we obtained a comprehensive view of the morphological and biochemical alterations occurring along the aging path of human RBCs in static or microgravity conditions as well as an overview on the priorities in the use of cellular resources (GSH and ATP) and on the metabolic strategies employed in starvation conditions.

The study in microgravity reveals the existence of adaptation and correlation mechanisms between the metabolic activity and the structural characteristics of the erythrocytes, driven and triggered by the environmental conditions. The mechanical stimulation provided by the 3D-clinostat acts as a major environmental accelerator of the RBCs’ aging and activates distinctive metabolic and morphological patterns compared to standard aging. In addition the stimulation is of sufficient magnitude to induce physiological phenomena, such as the expulsion of ATP, which is activated by overcoming a threshold of mechanical deformation^2,3^ and that, according to recent data^47^, could be stronger in the presence of environmental mechanical stresses or solicitations.

**Figure 8:**
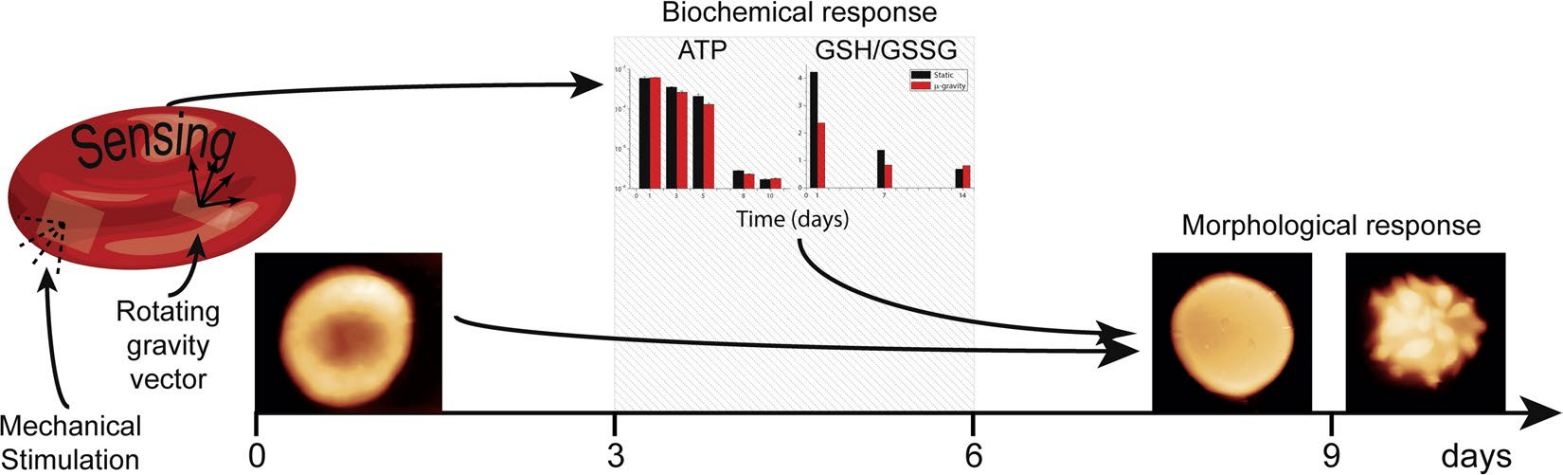
Model of the cellular response. Concept model for the RBCs sensing, integrating and translating environmental mechanical stimulation into morphological pattern along the aging.

The comparison between the RBCs response in microgravity and static conditions can be generalized in a new interpretative model. As sketched in Fig. 8, these cells undergo a rapid sensing of the environment that is initially translated into a metabolic adaptive response and subsequently fixed in structural and morphological alterations (therefore in modulations or alterations of functionality). Such a model, could represent a new paradigm to understand how erythrocytes translate and integrate over time the environmental stimulations.

A deeper understanding of such a sensing and translation mechanisms could also be important to elaborate new RBCs storage practices and to better understand the cellular and metabolic bases of some anomalies of clinical interest. Indeed, is important to note that the results of the exposure to microgravity are inherently dynamical and, in this perspective, they are more susceptible to comparison with the behaviour of cells in circulation.

## Materials and Methods

### Ethic Statement

Human blood was collected and used according to the ethical and safety regulations of the Country in which the samples were collected. In particular, the blood samples have been obtained, for the purpose of scientific research only, from healthy volunteers donors after written informed consensus, as required by Italian “National Bioethics Committee”. All the experiments, preparation methods and sample manipulation employed have been performed according to the highest standard of the medical practice and, in particular, in accordance to the relevant guidelines and regulations approved by the “Research Ethics and Bioethics Committee” of the Italian CNR.

### Samples Collection and Preparation

Blood samples were obtained from two healthy donors, using venipuncture into Vacutainers (Becton-Dickinson, Franklin Lakes, NJ, USA) containing ethylenediaminetetraacetic acid (EDTA) and were immediately centrifuged at 3000 rpm for 10 min at 4 °C. The yellowish supernatant (i.e. platelet-rich plasma) and the white coat on the pellet (i.e. leukocytes) were discarded, while an aliquot of plasma was stored at 4 °C for later use. After re-suspension and washing (4 times; 10% vol. fraction), the RBCs were stored under sterile conditions in glucose-free and calcium-free buffer solution (10 mM sodium phosphate, NaCl 140 mM, EDTA 1 mM) adjusted with NaOH to pH 7,4. A protease inhibitor (phenyl-methyl-sulfonyl-fluoride, 1 mM) was added to the RBCs’ suspension to avoid proteolytic degradation along the aging. The experiments were carried out at room temperature (20 +/− 1 °C) and at 20% of haematocrit.

The RBCs smears used for the AFM and the optical microscopy investigation were performed every 2–3 days as follows: an aliquot of the sample (at least 15 μl), was diluted 1:1 (v/v) in plasma. To avoid thermal stress, the plasma, stored at 4 °C, was allowed to reach room temperature before its addition to the samples. An aliquot of 5 μl of the plasma-enriched samples was smeared onto a commercial poly-L-lysine functionalized glass slide (Thermo Scientific, Menzel-Glaser). The dilution ratio 1:1 ensures homogeneous smears with an optimal density of cells over the whole glass slide. Each smear was performed in duplicate and air-dried. We verified that, properly stored, such smears remained stable over long time periods.

All the experiments involving microgravity have been repeated four times. In every experimental run, the samples and controls have been tested at different aging times for: lysis and Hb oxidation (every 1–2 days); the ATP (every 2–3 days); the GSH/GSSG (every 6–7 days). The revitalizations were performed 3 or 4 times for each experimental run and each of them produced an entirely new cascade of aging path.

The detailed description of the protocols employed is reported in the next sub-paragraphs.

### Microgravity Exposure System

Among the many available instrumentation and techniques to simulate microgravity conditions^25,48^, the 3D-clinostat configuration is employed by a large number of groups since it is particularly accurate when working with cells in suspension^12,49,50^. Specifically, the 3D-clinostat is preferable for the study of RBCs, since other similar instrumentation, such as the RPM, produce accelerations that can affect the trajectory of the cells’ motion, inducing a higher degree of perturbation^12^. The cells in the 3D-clinostat experience a free-fall, following very small spherical trajectories resulting in a good approximation of a microgravity environment^28,51^.

We used a custom machine developed in the LPMV-EPFL laboratories (Figure S1). The machine operates by rotating three-dimensionally the samples around two distinct and independent axes, with well-controlled speed and acceleration. The rotation over the two axes is controlled using two high-precision motors (Epos-2 by Maxon – MA, USA), capable of reliable and sustained rotation speeds varying from 0 to 10 rad/s.

We maintained a constant speed of 28 rpm on both axes via a computer user interface with dedicated control software. This resulted in a relatively fast complete 3D rotation, with period of 2,15 seconds: i.e. long, when compared to the scale of single biochemical events, but irrelevantly small in the time frame characteristic of the aging phenomenon. The choice of this speed rose from two conflicting requirements: to have a fast complete rotation and to maintain a small effect of centrifugal forces on the cells. By placing the cells in the rotational centre, the overall effect of this movement was that the RBCs experienced an average null gravity vector over the rotation period. In all other positions of the sample holder, the apparent gravity produced by centrifugal forces increased with the distance from this rotation centre. In our case, due to the size of the vials, the samples experienced a simulated gravity ranging from 0 g to less than 0.001 g. In this condition, the cell motion is expected to be a continuous free fall in liquid, following a spherical trajectory with a diameter ranging from 0 to few tens of microns, depending on the sample’s position and on the buffer’s viscosity.

In our experiments, we aliquoted the RBC samples dedicated to microgravity experiments in 0,25 ml single-use Eppendorf vials, which were carefully filled to avoid the presence of even tiny air bubbles. We placed these vials in the sample holder platform of the machine, and maintained them in continuous and constant rotation for the entire experiment.

### Conventional optical microscopy

The optical images were collected using an Olympus IX 70 inverted microscope. After performing an extensive mapping of each smear, with 400X magnification, the cells were counted and assigned to one of the four major morphological phenotypes: biconcave, crenated, spiculed (echinocytes) and spherical (spherocytes). To ensure a statistical relevance, at least 1000 cells per sample were counted.

### Erythrocytes’ lysis rate and haemoglobin oxidation state

To evaluate the percentage of cell lysis and the oxidation state of the intracellular and extracellular haemoglobin (Hb) we used a spectrophotometric method, slightly modifying the protocol proposed by Harboe^52^. The spectra were acquired using a double-beam spectrophotometer Jasco V-630, in the measurement range 350–700 nm where three well-defined peaks of the Hb spectra exist. We collected the Hb spectra each day of the experimental plan by the means of two different protocols:

- Extracellular Hb spectra: 60 μl from each sample were taken and diluted 20-fold in buffer solution, then centrifuged at 3200 rpm for 12 minutes in order to precipitate the RBCs. An 800 μl volume of the supernatant was collected and diluted 1:1 in buffer directly in the measurement cuvette.
- Intracellular Hb spectra: 6 μl from each sample was diluted 200-fold in bi-distilled water in order to lyse the cells, then the sample was centrifuged at 3200 rpm for 12 minutes; 1 ml of the supernatant was centrifuged at 11000 rpm for 11 minutes in order to separate and eliminate cell debris. An 800 μl volume of the supernatant was taken and diluted 1:1 with bi-distilled water directly in the measurement cuvette.

Errors due to the pipetting of the sample’s aliquots were evaluated at different aging times and resulted negligible. The percentage of lysis was measured simply by the ratio between the absorbance of the free Hb spectra at the maximum of the Soret peak and the same point in the intracellular spectra. Indeed, in our experimental conditions, the absence of bilirubin, plasma and free-cells in the solution allows to avoid the use of baseline correction or the 3-wavelenghts method^52^.

The Hb oxidation state was evaluated by monitoring the shift of the maximum of the Soret peak (which moves from 414 nm in the oxy-Hb towards lower wavelength according to the progressive formation of met-haemoglobin) in each measured spectra. All the data analyses were performed using the software package “Origin 8”.

### ATP measurements

An aliquot of the sample (typically 200 μl) was diluted one-fold in buffer solution and centrifuged at 3200 rpm for 12 minutes, then it was divided in two different fractions for the extracellular and intracellular measurements.

- For the extracellular ATP: a fraction of the supernatant (typically 250 μl) was centrifuged at 11000 rpm for 11 minutes to remove all the residual membrane debris in solution. A fraction (typically 200 μl) of the supernatant was used for the measurement of the extracellular ATP.
- For the intracellular ATP: the pellet was diluted 1:5 (v/v ratio) in perchloric acid 0,6 M; gently mixed and then centrifuged at 8000 rpm for 8 minutes to remove all the denatured proteins from the sample. A fraction of the supernatant (typically 80 μl) was neutralized in a new vial, by adding 6:1 (v/v ratio) potassium carbonate 2,5 M followed by one hour incubation in ice bath. Next, 240 μl of the supernatant were centrifuged at 11000 rpm for 11 minutes to eliminate any residual pellet. A 200 μl fraction of this supernatant was used for the measurement of the intracellular ATP.

The ATP content was detected using the “Cell Titer-Glo Luminescent Call Viability Assay” kit, from Promega. Each sample was measured twice in two different 96 multiwall plates, by adding 65 μl of the reagent to 65 μl of the sample and followed, according to the recommendations of the manufacturer, by 10 minutes incubation in a dark environment. The measurements were performed using a Wallac 1420 VICTOR3 V plate reader after 20, 30 and 40 minutes incubation. Each measured plate contained a standard calibration series of ATP that allowed to convert the luminescence values to ATP concentration. The ATP standards were prepared at the time of the measurements by progressive dilution of a batch 16,5 M ATP solution (Sigma).

The measured values of ATP were corrected for the dilution and the percentage of cell lysis (i.e. the data refer to the effective number of cells in solution). Moreover, to have homogenous results among the different experiments, the values of measured ATP were normalized to a RBCs solution at the standard haematocrit of 45%.

### Sample’s redox state: GSH/GSSG

Cold 10% metaphosphoric acid was carefully added (600 μl) to samples or standards (200 μl). After incubation (4 °C, 15 min) and centrifugation (20,000 × *g*, 15 min, 4 °C) supernatants were transferred into 1.5 ml propylene tubes (50 μl for the determination of GSH and 200 μl for the GSSG) and immediately stored at −80 °C.

- For the GSH assay: 1.0 ml of 0.1% EDTA in 0.1 M sodium hydrogenphosphate, pH 8.0, was added to 50 μl of the supernatant. Then, 300 μl of 0.1% EDTA in 0.1 M sodium hydrogenphosphate, and 20 μl of 0.1% ortho-phtaldialdehyde (OPA) in methanol, was added to 20 μl of this mixture. Well-capped tubes were incubated at 25 °C for 15 min in dark. The reaction mixture was then filtered through a 0.20 μm nylon filter of 4 mm diameter and stored at 4 °C.
- For the GSSG assay: a 200 μl portion of supernatant was incubated at 25 °C with 200 μl of 40 mM NEM for 25 min in dark to interact with GSH present in the sample. Then, 750 μl of 0.1 M NaOH was added. A 20 μl portion of this mixture was taken for measurement of GSSG, using the procedure followed for the GSH assay, except than 0.1 M NaOH was employed as diluent in place of the EDTA.

Chromatography of GSH and GSSG after their derivatization with OPA to form a stable, highly fluorescent tricyclic derivate^53^ was accomplished using isocratic elution performed with a HPLC Waters 600 pumps system and a AF Waters Online degasser on a X-Bridge C18, 5 μm, 4.6 × 150 mm column associated with a Symmetry C18, 3.9 × 20 mm guard (Waters, USA) at 37 °C. The mobile phase consisted of 15% methanol in 25 mM sodium hydrogenphosphate (v/v), pH 6.0. The flow rate was kept constant at 0.5 ml/min. The injection (50 μl) of samples or standards into the column was performed using a Waters 717 plus autosampler. The excitation/emission wavelengths were set to 350/420 nm in a Shimadzu RF-551 spectrofluorimetric detector. The instrument control and data acquisition were carried out using the Waters^®^ Millennium^® ^^32^ software. The concentration of GSH and GSSG in the samples was determined from the calibration curve, GSH and GSSG solutions were prepared daily in 1 mM hydrochloric acid and stored at 4 °C until use.

### RBCs’ rejuvenation

The rejuvenation procedure (also named revitalization) was carried out following a slightly modified De Venuto protocol^54^ at selected aging times, in order to evaluate, at different aging times, the behaviour of the fraction of metabolically active RBCs and to monitor their biochemical parameters.

To perform the rejuvenation protocol we employed a rejuvenation solution, called IPP: an isotonic solution composed by 10 mM inosine, 10 mM pyruvate, 75 mM sodium phosphate, 23 mM NaCl and 5 mM NaOH; with pH adjusted to 7,4.

A vial of sample was centrifuged for 12 minutes at 3000 rpm, the supernatant was discarded and the cells were suspended in IPP in ratio 1:5 (v/v), then incubated for 3 hours at 37 °C. After incubation the RBCs were washed twice, then suspended in the buffer solution with a final haematocrit of 20%. An aliquot of each sample (typically 200 μl) was used, immediately before and immediately after the rejuvenation procedure, to control the efficiency of the procedure.

### AFM and Roughness analysis

The AFM images were collected using a home-designed microscope customized for biological studies operated in contact mode under controlled environmental conditions (room temperature and constant 30% relative humidity). The measurements were performed in the weak repulsive regime of constant force with a probe force below 1 nN. Silicon Nitride Veeco MSCT probes (Camarillo, CA, USA) with 0,03 N/m elastic constant, asymmetric pyramidal shape and nominal tip radius of 10 nm were employed. The high resolution images were collected at a scanning speed of about 3–4 s/row and the reproducibility of data was carefully tested.

The high-resolution AFM images were massively employed for the measurement and analysis of the surface roughness.

The surface roughness of erythrocyte’s plasma membrane has been proved a sensitive parameter to evaluate the structural integrity and, as a consequence, the mechanical support that the cell skeleton can exert (a detailed discussion of the methodology employed for the measurement is reported in the reference)^5^. Here, however, we slightly modified the protocol in order to improve, especially in the range of the low values, the determination of the surface roughness, to increase the number of sampling x cell and to reduce the measurement error. The present method employs the free software Gwyddion (www.gwyddion.net), and consists in selecting several (typically more then 10) sampling areas on the cell membrane, all of fixed, 1 × 1 μm, size. After a background subtraction, all the residual morphological component was removed by fitting X and Y axis with a high grade polynomial (in the past, a first grade was employed). After testing the polynomial grade from 6^th^ to 9^th^ on a large number of RBC images with different morphological characteristics, we arbitrarily decided to use the 7^th^ grade fit as a best compromise between proper background subtraction and preservation of even the tiniest features on the cell membrane.

After the fitting, the surface roughness was measured using the formula:

1 $$Rrms=\frac{1}{(N-1)}\,\ast \,\sqrt{{\sum }_{1}^{N}{(Xi-Xm)}^{2}}$$

The present upgrade of the analysis protocol implies, in general, little quantitative differences in the measured roughness values compared to previously reported data^36^. In this sense, the data presently measured for the static samples can be used as an updated standard for the behaviour of the roughness in control RBCs along the aging.

### Data availability

All the data presented in this study is freely available from the corresponding author upon reasonable request

## Electronic supplementary material

Figure S1
