# Supplementary material for: Erythrocyte’s aging in microgravity highlights how environmental stimuli shape metabolism and morphology
Source: Sci Rep. 2018 Mar 27;8:5277. doi: 10.1038/s41598-018-22870-0 (PMC5869709; doi:10.1038/s41598-018-22870-0)
Supplement: Supplementary file 1 — Figure S1 [file 41598_2018_22870_MOESM1_ESM.pdf]

Supplementary materials: FIGURE S1.

Erythrocyte's aging in microgravity highlights how environmental stimuli shape metabolism and morphology

S. Dinarelli, G. Longo, G. Dietler, A. Francioso, L. Mosca, G. Pannitteri, G. Boumis, A. Bellelli and M. Girasole*

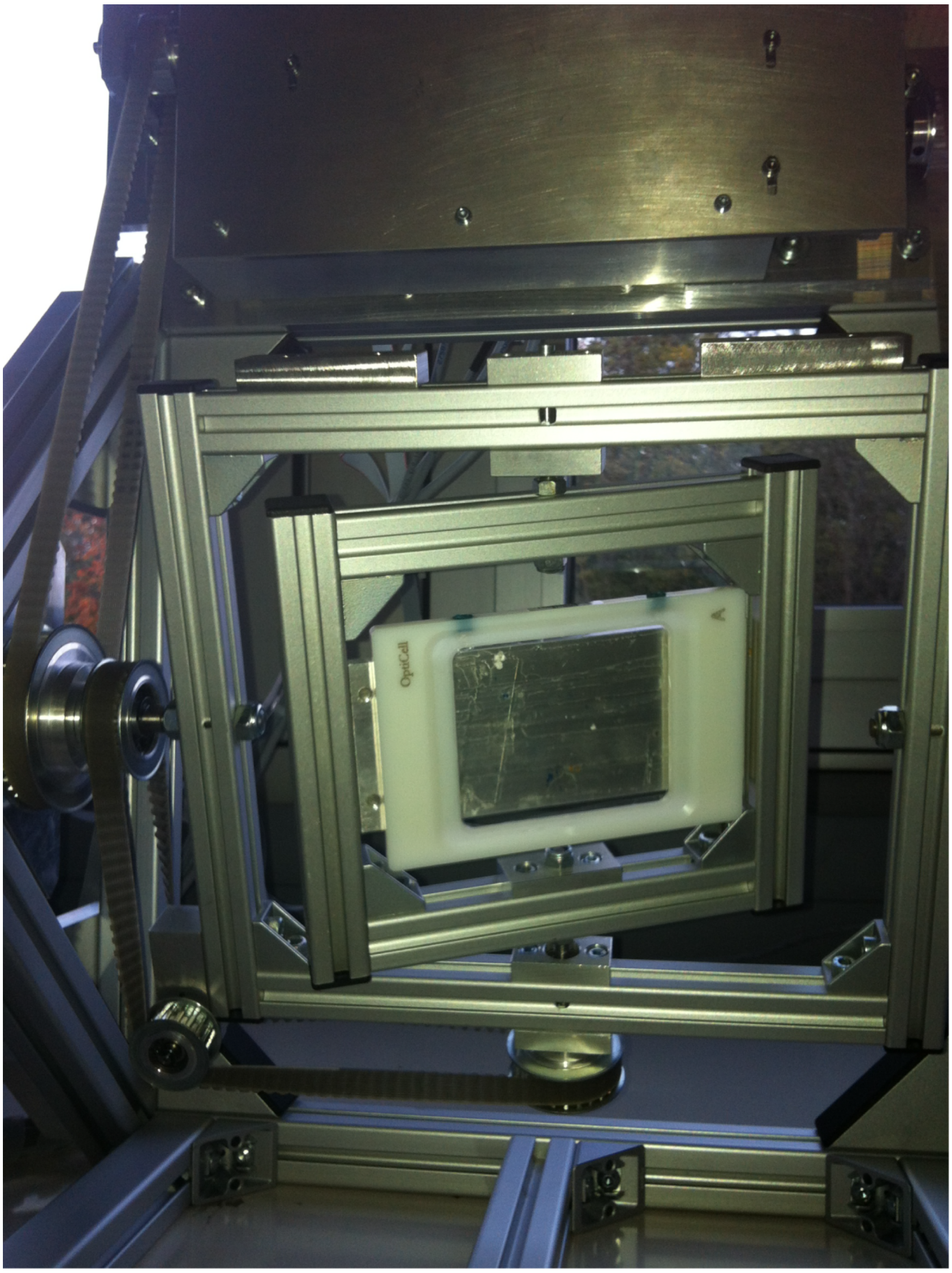

Figure S1. Image of the custom Random Positioning Machine. The dimensions of the 3D-clinostat are 50x50x70 cm with an inner and outer frame, which are independently controlled by the motors. The vials containing the samples were immobilized on the center of the platform located on the inner frame.
